# Efficacy and safety of CARTIGROW® in patients with articular cartilage defects of the knee joint: a four year prospective studys

**DOI:** 10.1007/s00264-022-05369-2

**Published:** 2022-03-28

**Authors:** Shirish Pathak, Deepak Chaudhary, K. Raghuveer Reddy, Kiran K. V. Acharya, Sanjay M. Desai

**Affiliations:** 1 Deenanath Mangeshkar Hospital & Research Centre, Pune, India; 2 Safdarjung Hospital and BLK Super Specialty Hospital, Delhi, India; 3 Sai Institute of Sport Injury & Arthroscopy, Hyderabad, Telangana India; 4 Kasturba Medical College & Hospital, Manipal, India

**Keywords:** Autologous chondrocyte implantation, Cartilage defects, Mid-term efficacy

## Abstract

**Introduction:**

Research shows autologous chondrocyte implantation (ACI) is a promising treatment for articular cartilage lesions. In this study, we assessed mid-term efficacy and safety of gel-based ACI or autologous adult live cultured chondrocytes (CARTIGROW®) implantation in patients with cartilage defects of the knee joint.

**Methods:**

In this prospective, open-label study, patients (19–38 years) with focal, international cartilage repair society grade III or IV articular cartilage defects of the knee joint were enroled at four centres across India from April 2015 to September 2015. Punch biopsy was conducted to harvest cartilage, from which chondrocytes were isolated and cultured, and the characterised chondrocytes were implanted into the cartilage defect. Key efficacy outcomes were assessed by quantitative changes in international knee documentation committee (IKDC), visual analogue scale (VAS) scores, and qualitative changes in magnetic resonance imaging at six months and four years from baseline.

**Results:**

Of the14 patients enroled in the study, all patients completed the six month follow-up and 11 completed the four year follow-up. The IKDC score improved significantly from 32.84 ± 9.25 at baseline to 67.49 ± 13.03 at six months (mean difference [MD] 34.66 ± 13.00, *p* < 0.0001) and to 60.18 ± 10.33 at four years (MD 28.21 ± 15.14, *p* = 0.0001). The VAS score reduced from 72.00 ± 14.40 at baseline to 16.64 ± 17.03 at six months (MD 55.36 ± 24.50, *p* < 0.0001) and further to 12.72 ± 9.05 at four years (MD 62.09 ± 10.66, *p* < 0.0001). All patients showed improvement on MRI of the knee joint. No adverse events were reported.

**Conclusion:**

Autologous adult live cultured chondrocytes (CARTIGROW®) implantation showed good mid-term efficacy in patients with cartilage defects of the knee joint with no side-effects.

## Introduction

Cartilage lesions of the knee are common findings in routine arthroscopy [1]. These may lead to pain, swelling, and functional impairment affecting the quality of life [2]. Cartilage injuries limit self-repair potential and often progress to diffuse osteoarthritis [2] that necessitates surgical intervention for symptomatic relief. Several surgical and non-surgical treatments for full-thickness cartilage and osteochondral articular lesions currently exist including microfracture, osteochondral autograft transfer, osteochondral allograft transplantation, and autologous chondrocyte implantation (ACI) [3]. However, choosing one treatment over the other remains debatable.

The role of ACI is established in managing cartilage defects, particularly in patients with limited treatment options or when non-surgical approaches have failed. In ACI, autologous chondrocytes harvested through cartilage biopsy are implanted into the debrided cartilage defect under a periosteal cover. Periosteum provides a waterproof covering for the implanted chondrocytes. Periosteal cambium may also contribute to healing by providing growth factors and mesenchymal stem cells that develop into chondrocytes [4]. Histologically, the tissues formed after ACI are superior to those formed by other techniques that repair cartilage. The primary goal of ACI is to heal and restore damaged surface of joints that could otherwise progress to osteoarthritis and improve overall joint function [5]. Evidence shows that ACI is more effective than bone marrow stimulation in reproducing cartilage [6]. Further, significant benefit of ACI over microfracture was reported in a randomised controlled trial [7]. ACI is financially more expensive than a simple bone marrow stimulation procedures like microfracture. But given that few people require a second repair or a total knee replacement, it is proven to be relatively more cost effective than major reconstructive surgeries at later date [8]. Its long-term durability of benefits for cartilage defects have been recently proven [9, 10]. It is currently the only method of reparative articular cartilage therapy approved by the United States Food and Drug Administration.

Older ACI techniques involve extensive surgical exposure and a potential risk for cellular leakage, graft detachment and graft hypertrophy. New generation ACI techniques use bioactive, resorbable materials to culture chondrocytes [11]. Most common method involves using collagen membrane to culture cells cut to exact proportions of the defect. Newer techniques are simpler than conventional techniques and do not use the periosteum. However, potential risks include loss of cells and membrane detachment [11]. Our study used CARTIGROW®, a gel-based ACI technique, which involves mixing ex vivo cultured chondrocytes with fibrin glue at implantation that facilitates even cell distribution in the lesion and helps their attachment to the cartilage defect [11]. In this study, we evaluated the efficacy and safety of CARTIGROW® technique for treating Indian patients with articular cartilage defects of the knee.

## Methods

### Study design and patient selection

This prospective, open-label, phase III study was conducted from April 2015 to September 2019 at four centres across India (Deenanath Mangeshkar Hospital & Research Centre, Pune; Vardhman Mahavir Medical College & Safdarjung Hospital, New Delhi; Krishna Institute of Medical Sciences, Secunderabad; Kasturba Medical College & Hospital, Manipal). Patients (19 to 38 years) with focal articular cartilage defects of the knee joint with grade III and IV severity per the International Cartilage Repair Society were enroled. Patients with intact meniscus, stable knee with normal alignment, and normal joint space with no inflammation or arthritic changes in the joint were eligible for the study. In patients with mal-aligned knee(s), corrective procedures were carried out either prior to or with biopsy. Patients with BMI > 35 kg/m^2^ and/or those with lesions of sizes < 1 cm^2^ or > 12 cm^2^ were excluded. Other key exclusion criteria included degenerative joint changes such as osteoarthritis, avascular necrosis and articular cartilage defects like kissing lesions, mal-aligned articulating joints, unstable articulating joint ligament and/or bone defects around the defective cartilage, and patients with inflammatory or rheumatoid arthritis. Patients whose cartilage cells did not grow sufficiently in vitro were also excluded from the study.

This study was conducted in accordance with the ethical principles that have their origin in the current Declaration of Helsinki (2013) and was consistent with The International Council for Harmonization of Technical Requirements for Pharmaceuticals for Human Use, Good Clinical Practice (GCP), Indian GCP guidelines issued by Central Drug Standard Control Organization, local regulations and ethical guidelines for biomedical research on human participants issued by Indian Council of Medical Research. The Drug Controller General of India, Central Drug Standard Control Organization under Ministry of Health & Family Welfare, Government of India approved this phase III clinical study. Patients were enroled only after obtaining approval from Ethics Committees of all four centres. The study was registered in the Clinical Trial Registry of India (CTRI/2015/04/005661). All patients and/or their legally acceptable representatives provided informed consent before participation.

### Study procedure

**Fig. 1:**
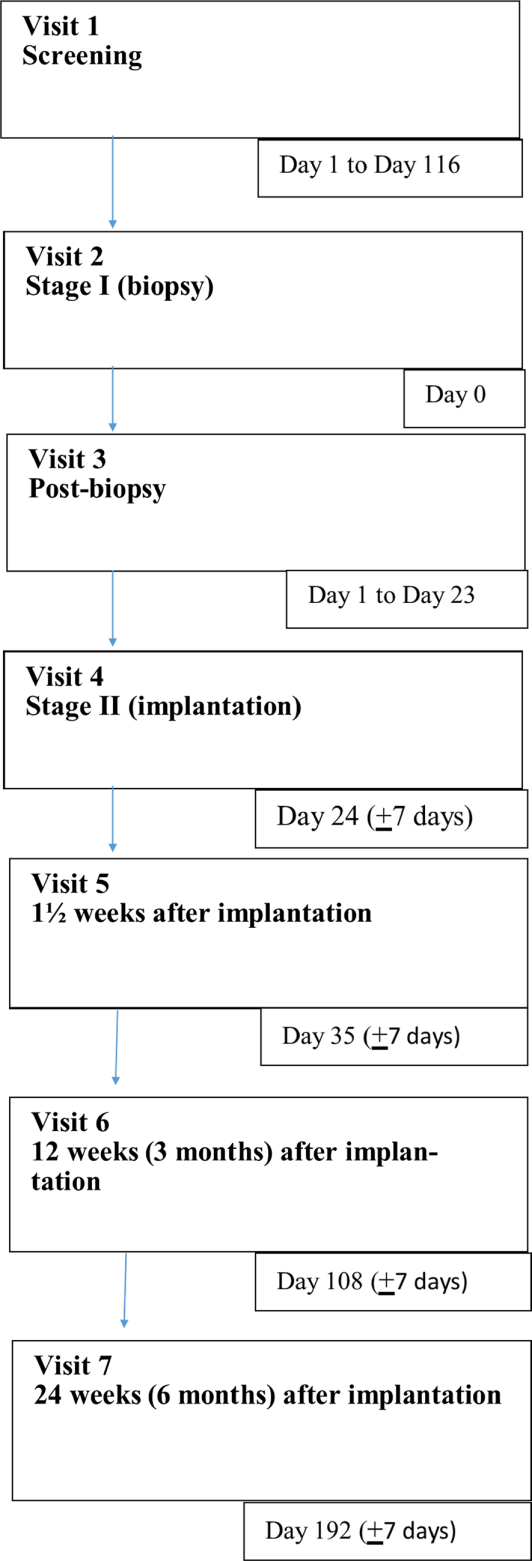
Flowchart of study visits

This study involved seven visits, each for screening, biopsy, pre-implantation, implantation, and three follow-up visits at 1.5 weeks, three months and six months (Fig. 1). The follow-up visit at 4 years was not mandatory (voluntary visit), and hence, the patient data of those reporting to clinic for routine care were collected. After initial patient screening, CARTIGROW® procedure was conducted in two stages. In the first stage, arthroscopy was performed to evaluate the osteochondral defect. Loose bodies secondary to trauma or osteochondritis dissecans were removed during the procedure. A full-thickness articular cartilage punch biopsy was performed to harvest hexagonal osteochondral cylinders (approximately 6–8 mm in size) with the subchondral bone. All unstable and damaged cartilage was removed with utmost care to avoid penetration into the subchondral bone. Cartilage specimen(s) were then sent to a GMP-certified cell culture laboratory (Regrow Biosciences Pvt. Ltd.) in a sterile container with culture medium. Harvested cells from the biopsy were then processed for three to four weeks in the laboratory to achieve a uniform suspension (CARTIGROW®). If cells could not be grown or cultured within seven days for any patient, they were considered a “screen failure.” The final product was packaged in cold chain (2–8°C) and transported to the hospital for implantation. In the second stage, arthrotomy was performed and CARTIGROW® was implanted directly onto the defect while maintaining a gravity eliminating position parallel to the floor to ensure that the implant did not overflow into the surrounding areas. Stability of the implant was assessed by moving the knee from full extension to flexion for ten cycles. Skin and muscle defects were closed in layers and a compression dressing was applied.

### Rehabilitation program

Patients followed a post-operative rehabilitation program that included non-weight bearing exercises with walker immediately post-ACI to four weeks after ACI gradually followed by partial weight-bearing exercises at five to eight weeks. Using continuous passive motion (CPM) machine, 140° of motion range was achieved at eight weeks after ACI. Patients were also allowed early mobilisation for ranges 0–90° with CPM machine. Muscle strengthening exercise: four point exercise, isometric exercise, hamstring exercise, and squatting exercise were advised; and 12 weeks after ACI, patients could perform stationary bike activities without resistance. Patients would start walking lightly at 13 weeks and jogging at six months followed by high-intensity exercises and sports activities, nine months after ACI. Subjects were advised to avoid any non-drug therapies like massage, acupuncture, acupressure, or any other method of joint manipulation for the affected joint during the study period. Deviations from the rehabilitation program were at the investigator’s discretion based on patient’s condition.

### Study outcomes

Efficacy endpoints were change in the International Knee Documentation Committee (IKDC) knee examination score and the VAS score from baseline to six months and four years. The other endpoints of improvement in magnetic resonance imaging (MRI) from baseline to six months and four years were subjectively/qualitatively assessed by an independent radiologist. The IKDC score is a patient-completed questionnaire with responses categorised on ordinal scale. Total possible score ranges from 0 to 100 where 100 represents no limitation with daily or sporting activities and absence of symptoms [12]. Pain VAS provides one-dimensional measure of pain intensity on a vertical or horizontal continuous scale 10 cm in length, anchored by two verbal descriptors, one for each symptom extreme. A higher score indicates greater pain intensity. MRI examinations were performed at not less than 1.5-T and evaluated qualitatively to assess the regeneration of articular cartilage at the defect site. Morphological scoring was done with a modified magnetic resonance observation of the cartilage repair tissue.

The magnetic resonance observation of cartilage repair tissue (MOCART) score was done by single, independent, experienced musculoskeletal radiologist at four years who was blinded to the clinical details and clinical outcome assessment [13]. Safety assessments (physical examination, vital signs and laboratory assessments) were performed at biopsy visit until the follow-up visit at six months.

### Statistical methods

Continuous variables are presented as means and standard deviations or medians with 25th and 75th percentiles for asymmetrical distributions and were compared using paired *t* test. Categorical variables are summarised as frequency counts and their percentages. Statistical analysis of the data was performed using SAS® version 9.3. All inferential analyses were performed at 5% level of significance for two-sided alternatives.

### Sample size calculation

A total of 13 patients were required to test the hypothesis with 80% power at *p* = 0.05 based on the mean reduction of VAS score of 27.19, and standard deviation of 31.33 reported in previous literature [14]. After considering a 10% drop-out rate, our sample size was 14 patients.

## Results

**Fig. 2:**
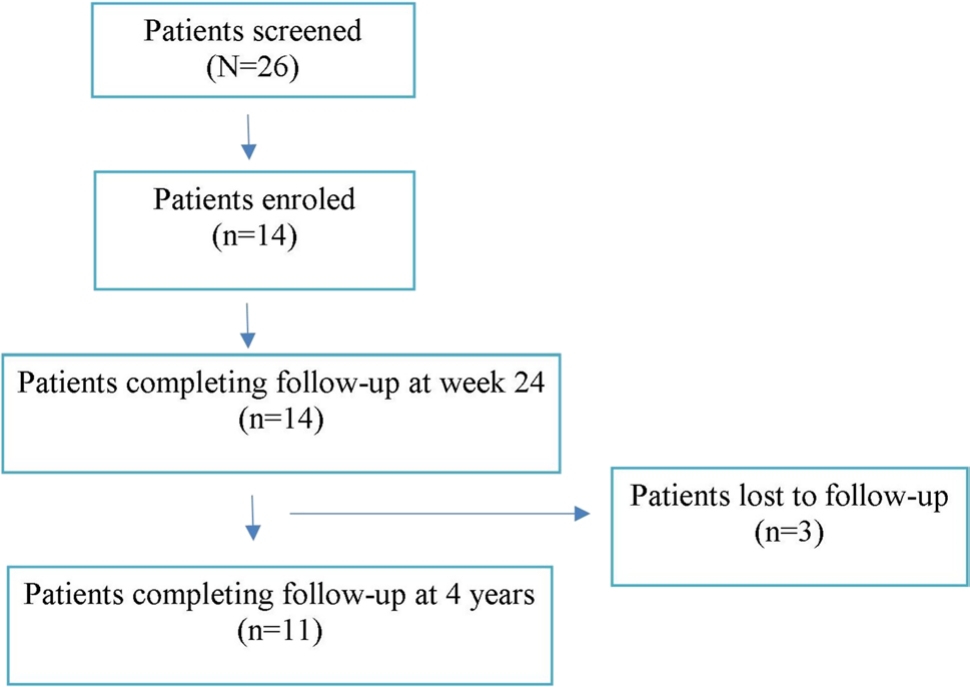
Patient disposition

Of the 26 screened patients, 14 were enroled and completed the study at six months. The mean age of patients was 29.57 ± 5.83 years. Three patients were lost to follow-up and could not complete the study visit at four years (Fig. 2). The mean period of follow-up was 53.18 months.

Of those recruited, ten were men and four women. Participants had a mean weight of 71.15 kg, height of 166.29 cm, and BMI of 25.66 kg/m^2^ at baseline. Details regarding the location of the defect and the range of defect size for all 14 patients are given in Table 1.

**Table 1:** Background data of enroled patients

Parameter	Value (*n* = 14)
Age (years) (mean ± SD)	29.57 ± 5.83
Sex	
Male	10 (71.4)
BMI (kg/m^2^)	25.66 kg/m^2^
Defect location (*n*)	
Left knee LFC	4
Left knee MFC	4
Right knee LFC	2
Right knee MFC	4
Defect size range (cm^2^)	1.21–10.14

*LFC* lateral femoral condyle, *MFC* medial femoral condyle

Data presented in (*n*) or mean ± SD

### Safety assessment

No adverse events or serious adverse events were reported at the end of six months and during the voluntary follow-up at four years.

### IKDC score

The IKDC score improved from baseline (32.84 ± 9.25) to 67.49 ± 13.03 at six months (67.49 ± 13.03) (*p* < 0.001) but reduced at four year follow-up (60.18 ± 10.33), but was still statistically significant compared to baseline (*p* = 0.0001) (Table 2).

**Table 2:** Changes in IKDC score and VAS score

Score	Baseline	6 months	4 years
IKDC score	32.84 ± 9.25	67.49 ± 13.03	60.18 ± 10.33
Mean difference	–	34.66 ± 13.00	28.21 ± 15.14
*p* value		< 0.001	0.0001
VAS score	72.00 ± 14.40	16.64 ± 17.03	12.72 ± 9.05
Mean difference	-	-55.36 ± 24.49	62.09 ± 10.66
*p* value		< 0.0001	< 0.0001

*IKDC* International Knee Documentation Committee, *VAS* visual analogue scale

Data presented as mean ± SD

### VAS score

Mean VAS score reduced significantly at 24 weeks vs baseline (72.00 ± 14.40 vs 16.64 ± 17.03, mean reduction: − 55.36 ± 24.49, *p* < 0.0001) that further reduced at four years (12.72 ± 8.62) suggesting statistically significant reduction in pain symptoms (*p* = 0.0001) (Table 2).

### MRI findings

**Fig. 3:**
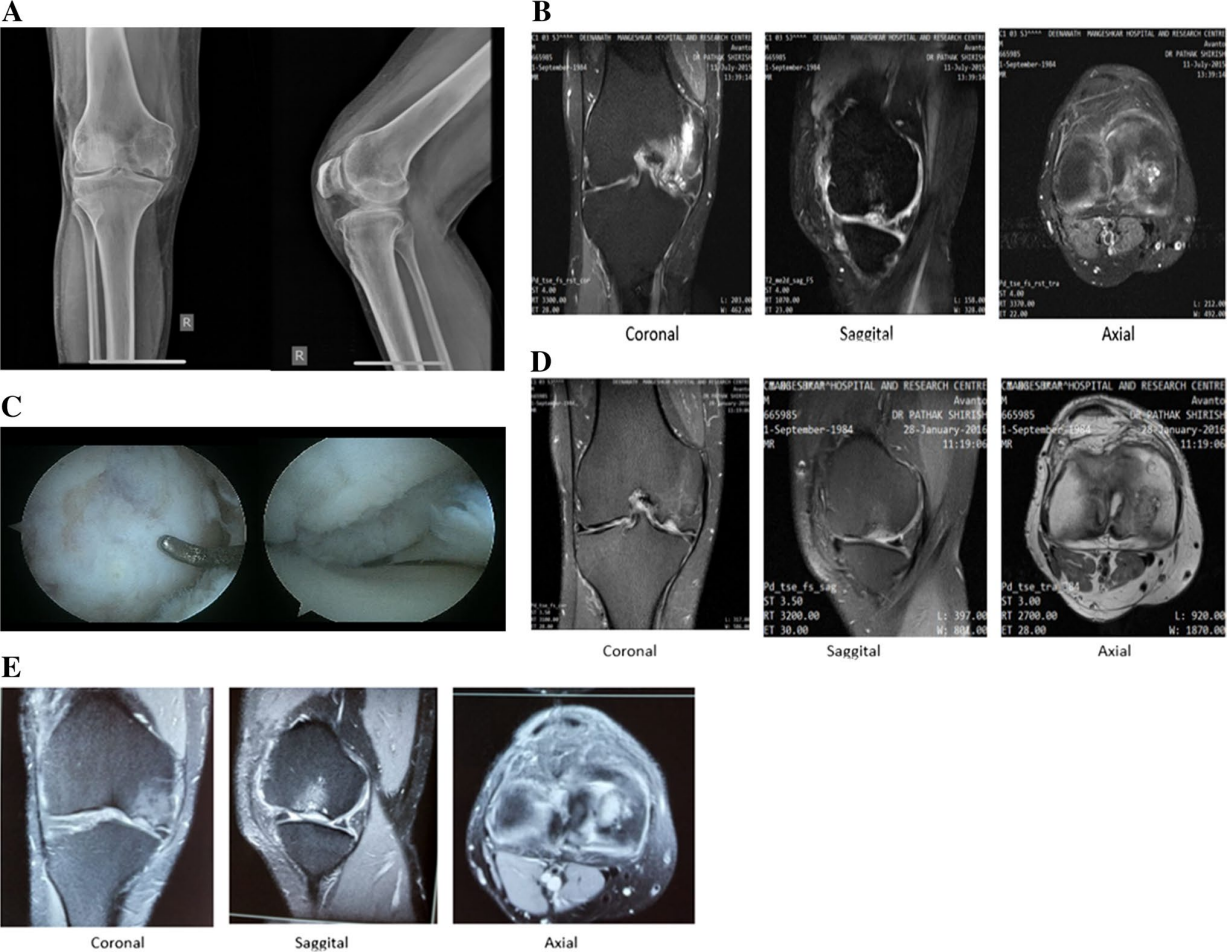
Pre-operative and post-operative clinical evaluations showing new cartilage formation in patient 1. **A** Pre-operative X-ray of the right knee AP and lateral showing cartilage defect on right medial femoral condyle. **B** Pre-operative MRI images of same the patient with PD-FS/T 2 coronal, sagittal and axial sections showing grade 4 osteochondral defect over medial femoral condyle. **C** Arthroscopic image showing ICRS grade 4 over medial femoral condyle. **D**, **E** Post-operative MRI images at 6 months and 4 years of the same patient with PD-FS coronal, sagittal and axial sections showing good healing of cartilage defect post ACI, respectively

**Fig. 4:**
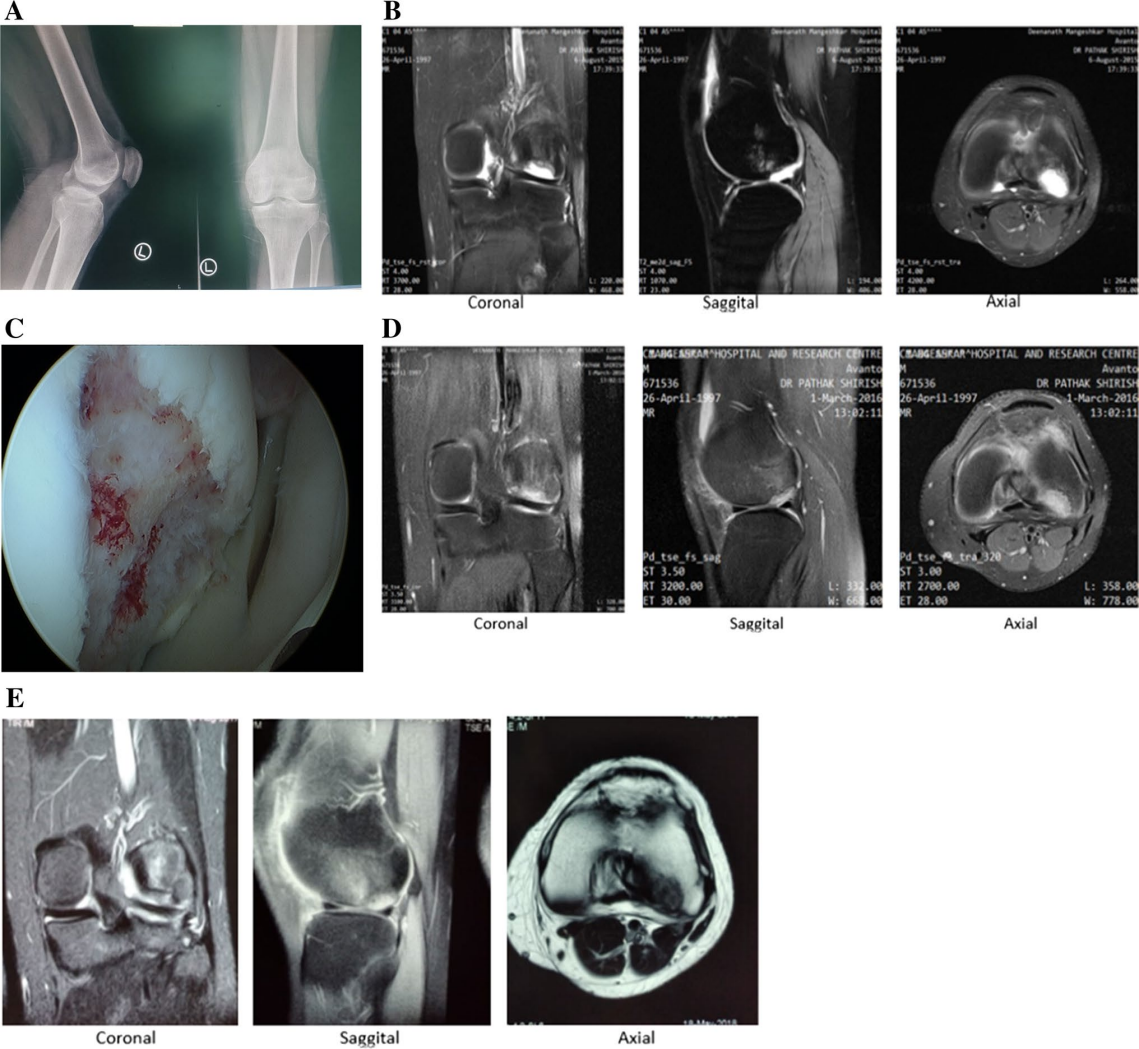
Pre-operative and post-operative clinical evaluations showing new cartilage formation in patient 2. **A** Preoperative X-ray of the left knee AP and lateral showing cartilage defect on left lateral femoral condyle. **B** Pre-operative MRI images of the same patient with PD-FS/T 2 coronal, sagittal and axial sections showing grade4 osteochondral defect over lateral femoral condyle. **C** Arthroscopic image showing ICRS grade 4 over lateral femoral condyle. **D**, **E** Post-operative MRI images at 6 months and 4 years of the same patient with PD-FS coronal, sagittal and axial sections showing good healing of cartilage defect post ACI, respectively

Definite improvement from baseline MRI was noted in the follow-up MRI conducted at four years for seven patients. The MOCART was performed at four years to evaluate cartilage regeneration. The defect was filled completely with regenerated articular cartilage in 4/7 (57%) patients compared to baseline. Optimal cartilage interface was seen in 5/7 (71%) patients with 4/7 (57%) them showing intact cartilaginous integrity (Table 3). Additionally, regenerated cartilage showed continuity with the surrounding articular cartilage with no fissure, cleft formation or any other irregularities and no signs of infections in surrounding tissues with mean MOCART score of 60.71 ± 26.65 (Fig. 3). Figure 4 shows pre-operative, intra-operative and post-operative arthroscopy images showing the defect before and repair of defect after the treatment. Overall, no patients required any surgeries for treating cartilage repair, or hyaluronic acid/steroids at six months (*n* = 14) or four years post-treatment (*n* = 7).

**Table 3:** Post-operative MOCART score of patients at 4 years (*n* = 7)

Parameter	Item	Points							Patients *N* (%)
		Patient 1	Patient 2	Patient 3	Patient 4	Patient 5	Patient 6	Patient 7	
Defect fill	Bone exposed	–	–	–	–	–	–	–	–
	Incomplete < 50%	–	–	5	-	-	5	–	2 (28.7)
	Incomplete > 50%	–	–	–	–	–	–	–	–
	Complete	20	20	–	20	20	–	–	4 (57.1)
	Hypertrophy	–	–	–	–	–	–	15	1 (14.3)
Cartilage interface	Complete	15	15	–	15	15	–	15	5 (71.4)
	Border visible	–	–	–	–	–	–	–	–
	Defect visible < 50%	–	–	–	–	–	–	5	2 (28.7)
	Defect visible > 50%	–	–	–	–	–	–	–	–
Surface of repair tissue	Intact	10	10	–	10	10	–	–	4 (57.1)
	Damaged < 50%	–	–	–	–	–	–	5	2 (28.7)
–	Damaged > 50%	–	–	–	–	–	0	–	1 (14.3)
Structure of repair tissue	Homogeneous	5	5	–	5	5	–	5	5 (71.4)
	Inhomogeneous	–	–	0	–	–	0	–	2 (28.7)
Signal intensity	Normal	–	30	–	–	30	-	–	2 (28.7)
	Nearly normal	10	–	10	10	–	10	10	5 (71.4)
	Abnormal	–	–	–	–	–	–	–	–
Subchondral lamina	Intact	5	5	–	5	–	–	5	4 (57.1)
	Not intact	–	–	0	-	0	0	–	3 (42.9)
Subchondral bone	Intact	5	–	–	–	–	–	5	3 (42.9)
	Abnormal	–	0	0	–	0	0	–	4 (57.1)
Adhesions	Yes	5	–	–	–	–	–	–	1 (14.3)
	No	–	0	0	0	0	0	0	6 (85.7)
Effusion	No	5	5	–	–	–	–	5	4 (57.1)
–	Yes	–	–	0	–	0	0	–	3 (42.9)
Total score		75	90	20	75	80	20	65	–

*MOCART* magnetic resonance observation of cartilage repair tissue

Data presented in (*n*) or mean ± SD

## Discussion

In this study, patients who underwent ACI procedure for treating their articular cartilage defects showed significant improvements in their IKDC and VAS scores that was further substantiated by MRI results showing significant reduction in the lesion size at six months and four years compared to baseline. Improvements in the VAS and IKDC scores were sustained even after four years of the procedure suggesting mid-term clinical benefits and symptomatic relief. Other clinical studies have also validated its efficacy and sustainability over time [4, 15].

The aim of clinical therapy is to regenerate hyaline cartilage that can integrate with surrounding cartilages and the underlying bone, thereby restoring normal knee function. Currently available treatment modalities have variable success rates. Microfracture is most commonly used treatment modality for treating articular cartilage defects that involve creating small holes perpendicular to the subchondral bone plate allowing bleeding into the defect. Microfracture leads to a fibrous-fibrohyaline unstructured repair tissue that lacks biomechanical and viscoelastic features of hyaline cartilage showing short-term improvement of symptoms. However, this is usually followed by repair tissue failure and gradual deterioration to osteoarthritis, and symptoms return after two to five years [16]. Later-on, osteochondral autografts, allografts or mosaicplasty aim to regenerate physiologically similar cartilages. Recent research has shown the advantages of chondrocyte implantation in treating cartilage defects [17, 18]. However, ACI is a simple technique with fewer donor site complications in treating full-thickness cartilage lesions of the knee with durability by maintaining good clinical results for longer times [4].

Significant subjective and objective clinical improvements were observed with ACI up to ten years following the treatment in a study where among patients with isolated femoral condyle lesions, most patients (84–90%) graded the benefits/experiences to be good and/or excellent [19]. Haris et al. conducted a long-term study of ACI in patients with lesions in patella, trochlea and the patellofemoral joint. Over 90% of the treated patients reported being satisfied with the procedure and showed consistent clinical outcomes 12 years after the implantation [15]. Efficacy and persistence of clinical effects of ACI is also documented in large full-thickness cartilage and osteochondral lesions of the knee. A study evaluated 341 patients with knee cartilage injuries who were treated with ACI. Long-term follow-up of those patients suggested sustained clinical and functional benefits even after ten to 20 years post implantation [4]. Long-term follow-up study also demonstrated excellent results with ACI in patients with symptomatic cartilage defects of the knee. These results were durable even 20 years after surgery in more than 70% patients who underwent ACI treatment [20]. Similar results were seen in our study with clinical benefits being sustained at four years following the procedure.

Dalal et al. in their study showed improved or stable MOCART score with mean score of 63 at 57 weeks in most patients after treatment [21]. Genovese et al. reported mean MOCART score of 65 at five years post-ACI [22]. Niemeyer et al. reported mean MOCART score of 50 at ten years post-transplantation [23]. Our results are in line with these studies. Overall, the MRI findings suggested good cartilage repair, no signs of infection or effusion in joint cavity. Moreover, results should be compared cautiously as sometimes patients with poor MOCART score can have better functional activity owing to variability in the procedure by surgeons and adherence to rehabilitation protocol and physiotherapy.

Other techniques are used for treating articular cartilage defects of the knee joint, and they have showed comparable clinical outcomes [18]. An advantage of CARTIGROW® technology is that since it is a gel-based ACI technique, it can maintain the shape of articulation creating a three-dimensional structure approximately five minutes after injection. In case of defects along the chondral margin, fibrin aids in maintaining the shape of the graft as per the articulation. Fibrin also helps control bleeding in the bone [24]. It can cover large area about 15–18 cm^2^ of defect and allow for convexing of the surface. Gel ACI offers a successful treatment option for both small and large cartilage defects. Implanted cells are highly characterised chondrocytes that show better structural repair compared to uncharacterised cells that may lose their ability to re-express articular cartilage phenotype in vivo [6, 25]. Better regenerative potential of isolated and characterised chondrocytes used in our product may have resulted in complete filling of cartilage defect by regenerated cartilage without any irregularity as indicated by MRI findings in 50% of our participants. Our study limitation is the small sample size that was however approved for clinical trial. In addition, patients with comorbid conditions such as diabetes mellitus, renal and hepatic dysfunction were excluded from the study. Based on safety and efficacy profiles from the phase three study, CARTIGROW® is approved by the Drug Controller General of India, Central Drugs Standard Control Organization of the Government of India.

## Conclusion

Our study shows that gel-based ACI alleviates pain and improves functional activity with no side-effects in patients with articular cartilage defects of the knee. Overall, CARTIGROW® showed good mid-term efficacy; however, substantiating these results in real clinical setting would be of future interest.

## Data Availability

All data is provided with this manuscript.
