# Correction to: Efficacy and safety of CARTIGROW® in patients with articular cartilage defects of the knee joint: a four year prospective studys

**DOI:** 10.1007/s00264-023-05845-3

**Published:** 2023-05-29

**Authors:** Shirish Pathak, Deepak Chaudhary, K. Raghuveer Reddy, Kiran K. V. Acharya, Sanjay M. Desai

**Affiliations:** 1 Deenanath Mangeshkar Hospital & Research Centre, Pune, India; 2 Safdarjung Hospital and BLK Super Specialty Hospital, Delhi, India; 3 Sai Institute of Sport Injury & Arthroscopy, Hyderabad, Telangana India; 4 Kasturba Medical College & Hospital, Manipal, India

**Correction to: International Orthopaedics (2022) 46:1313–1321**

**https://doi.org/10.1007/s00264-022-05369-2**

Clinical Trial Registry of India (CTRI) number mentioned in the paper is incorrect. The correct CTRI number is CTRI/2015/04/005661.

The original article has been corrected.

